# The evidence landscape for direct-from-urine antibiotic susceptibility testing (AST) for urinary tract infection (UTI): a scoping review

**DOI:** 10.1093/jacamr/dlag170

**Published:** 2026-08-25

**Authors:** Atiyeh Asadpour, Richa Sharma, Jana Suklan, Stephen P Kidd, Julie Hart, Alexander Daniel Edwards, Sarah Helen Needs

**Affiliations:** Reading School of Pharmacy, University of Reading, Reading, UK; Reading School of Pharmacy, University of Reading, Reading, UK; NIHR HealthTech Research Centre in Diagnostic and Technology Evaluation, Newcastle University, Newcastle upon Tyne, UK; Microbiology Department, Basingstoke & North Hampshire Hospital, Hampshire Hospitals NHS Foundation Trust, Basingstoke, UK; Molecular Diagnostics Hub, Royal Hampshire County Hospital, Hampshire Hospitals NHS Foundation Trust, Winchester, UK; Reading School of Pharmacy, University of Reading, Reading, UK; Henley Business School, University of Reading, Reading, UK; School of Electronics and Computing, University of Southampton, Southampton, UK; Reading School of Pharmacy, University of Reading, Reading, UK

## Abstract

**Background:**

Antimicrobial susceptibility tests (AST) are vital to guide antibiotic selection to treat bacterial infections, including urinary tract infections (UTI). Current laboratory AST takes at least 2 days. Direct testing of urine samples could speed up testing, with rapid reporting aiding timely treatment.

**Method:**

A scoping review was conducted to identify studies assessing the accuracy of direct-from-urine AST, and map the breadth of evidence. Studies that measured overall categorical agreement (CA) compared to a reference method were selected, and the reported accuracy, the type of AST method and study methodology assessed.

**Results:**

In total, 33 studies were identified that met the inclusion criteria (1976–2024). These reported overall CA for direct-from-urine AST ranging from 78.7% to 100% compared to a reference method. These studies varied in the AST method used; in urine sample collection, origin and processing; in the antibiotics evaluated; in study design and data analysis. The most common AST method evaluated was direct-from-urine disc diffusion. Most studies reported accuracy only for samples containing a single Gram-negative organism.

**Conclusion:**

Although increasing numbers of studies report the accuracy of direct-from-urine AST, heterogeneity prevented systematic comparison of methods or detailed meta-analysis. Many studies reported overall accuracy >90% CA for urine samples containing a single Gram-negative organism across several direct-from-urine AST methods, offering potential time saving compared with current methods. However, as single organism samples represent only a subset of urines taken from UTI patients, additional studies—that include analysis of samples containing mixed cultures and Gram-positive isolates—are still required.

## Introduction

Antimicrobial susceptibility testing (AST) is an important step in infection management, providing vital information to guide the appropriate and effective treatment of bacterial infections. The current methods recommended by national^1^ and international guidelines^2^ take at least 48 hours, requiring overnight plating and pathogen identification, followed by AST performed on a pure bacterial isolate. National and international action plans to combat antimicrobial resistance highlight the need for innovation to develop new approaches to diagnose infections and inform appropriate infection management.^3^

New emerging methods—often incorporating novel technology—offer the promise of more rapid AST to inform antibiotic prescriptions. A review of commercial and non-commercial rapid phenotypic methods and microbiological testing technologies identified a range of different methods with faster sample-to-result times, in some cases under 1 hour.^4^ Most of these rapid methods use a direct-from-sample approach, avoiding the initial overnight plate culture to obtain an isolate, thereby cutting time to result by at least 1 day. This approach has been used extensively for positive blood cultures, meaning rapid results can decrease the time for effective antibiotic therapy for life threatening infections.^5^ The EUCAST rapid AST (RAST) method uses a modified disc-diffusion method directly from positive blood cultures to provide specific breakpoint results at 4, 6 and 8 h of incubation.^6,7^ Direct testing of positive blood cultures has also been a major focus of commercially available rapid AST technologies.^4^ It should be noted that blood cultures may require 24 h or more incubation time to culture positivity due to the low concentration of pathogens present in the initial blood sample. Whereas the EUCAST RAST method has been validated for positive blood cultures, there is no approved reference method for directly performing AST on any other sample types, including urine. Other sample types—such as urine—that have a higher bacterial load, may not require an incubation stage, offering the potential for rapid direct-from-sample testing. Several factors that are known to affect AST may present technical barriers to direct urine testing—such as variation in inoculum cell density, interference by urine sample matrix components and polymicrobial mixtures—so a comprehensive validation of these methods remains important.

The clinical urgency for rapid AST results for blood infections has driven commercial product innovation, however, providing the correct antibiotic treatment for urinary tract infections (UTIs) sooner also represents a significant concern for both clinicians and patients. The cost of treating UTIs in England between 2023 and 2024 was £604 million.^8^ UTIs are one of the most common reasons for antibiotic prescribing.^9^ Antibiotic resistance is a challenge for clinicians treating UTIs,^10^ as prescribing antibiotics with higher levels of resistance in the community leads to increased treatment failure.^11^ Not all patients in the community will have a urine sample sent to culture; of 743 350 UTI episodes in GP practices between 2015 and 2022, 50.8% had a urine test.^12^ With some high throughput methods, rapid AST for urine culture has the potential to increase the number of samples tested, as well as report results faster. As antimicrobial resistance in UTIs is increasing, more patients are experiencing more frequent and recurrent infections, driving the demand for more rapid AST to inform more targeted antimicrobial prescribing, which could be addressed by direct-from-urine testing.

To help drive acceptance of direct-from-urine AST methods beyond RAST for positive blood cultures, there is a need for a comprehensive analysis of the available evidence. Previous reviews have described efforts to achieve faster results using direct-from-sample methods, including direct-from-urine AST.^4^ Other reviews have focused on emerging technology for rapid AST, such as microfluidics,^13^ or on UTI diagnosis.^14^ To date, a scoping review mapping the evidence base for direct-from-urine AST has not been presented.

Here, for the first time, we conduct a scoping review that aims to map the current evidence base for direct-from-urine AST for patients with suspected UTI. We searched for studies that report the accuracy, reported time to result and the variety of analytical methods and approaches used of direct-from-urine methods—including those based on modified disc diffusion and broth microdilution, and using commercially available AST technologies—benchmarked against established reference standards. We also explore technical challenges and methodological considerations in performing direct-from-urine AST, and review how studies were designed to evaluate the accuracy of such tests.

Performing this scoping review to establish the extent of evidence reporting the accuracy of direct-from-urine AST is important because direct-from-urine testing offers great potential to reduce turnaround time and alleviate the workload associated with conventional culture methods. Although faster results have the potential to improve antibiotic prescribing, enable earlier appropriate treatment and reduce symptoms progression and antimicrobial resistance, it is important to understand the breadth and quality of the evidence base for direct-from-urine testing before such methods could be adopted.

## Methods

The protocol for this review was not prospectively registered in a public database.

### Eligibility criteria

Table 1 outlines the inclusion criteria following the PICOS criteria.

**Table 1.** Inclusion criteria (PICOS)

Population	Sample types included clinical urine samples identified as suspicion of UTI and/or sent for microbiological analysis. No restriction on age, sex, study date or geography.
Intervention	Direct-from-urine AST.
Comparator	Any AST reference method including broth microdilution and disc diffusion or commercially available *in vitro* diagnostic approved method.
Outcome	Measure of accuracy including CA.
Study design	Diagnostic test accuracy study.

### Types of study

All study types were included.

### Index test

The index test was required to output a measure of phenotypic or culture-based antibiotic susceptibility or resistance directly from a human urine sample, without an initial overnight agar plate culture step. This contrasts with current methods in which an overnight agar plate streaked with urine is followed by picking one or more colonies and AST being performed on these isolates. We included index tests that allowed for direct-from-urine AST with limited sample processing or handling steps performed on the urine. We only included nucleic acid-based tests where the output of the measurement was molecular/genetic but followed a growth period of bacteria in the presence of antibiotics. We therefore excluded studies that used molecular methods to detect specific resistance genes or genetic features associated with resistance, as these do not measure antibiotic susceptibility. The index test may take the form of a protocol, method or a device. We included both commercial and non-commercially available tests including prototype and proof-of-concept devices or protocols.

### Comparator

Included studies compared this index test to conventional routine phenotypic antimicrobial susceptibility tests. Comparators considered included reference methods including disc diffusion, broth microdilution and agar dilution. We also included commercially available AST systems as comparators, such as Vitek^®^ 2 (from bioMérieux), Sensititre^TM^ (from Thermo Fisher Scientific) or BD Phoenix^TM^ (from Becton Dickinson), which are used widely in routine clinical diagnostic and/or research microbiology laboratories.

### Primary outcome measure

- Accuracy of direct-from-urine AST

### Secondary outcome measures

- Study design
- Analytical method
- Assay time

### Search strategy

We performed electronic searches in the databases PubMed, Scopus and Web of Science. The main search terms were ‘direct urine’,’ UTI’, ‘bacteriuria’, ‘urine specimen’, ‘urine culture’, ‘direct antibiotic susceptibility testing’, ‘AST’, ‘direct AST’, ‘disc diffusion’, ‘direct disc diffusion’, ‘Flexicult’ and ‘DST’. We searched for literature published up to 29 October 2024. The search was conducted without any date or geographical restrictions. The study was restricted to English language studies.

### Selection of studies

**Figure 1.**
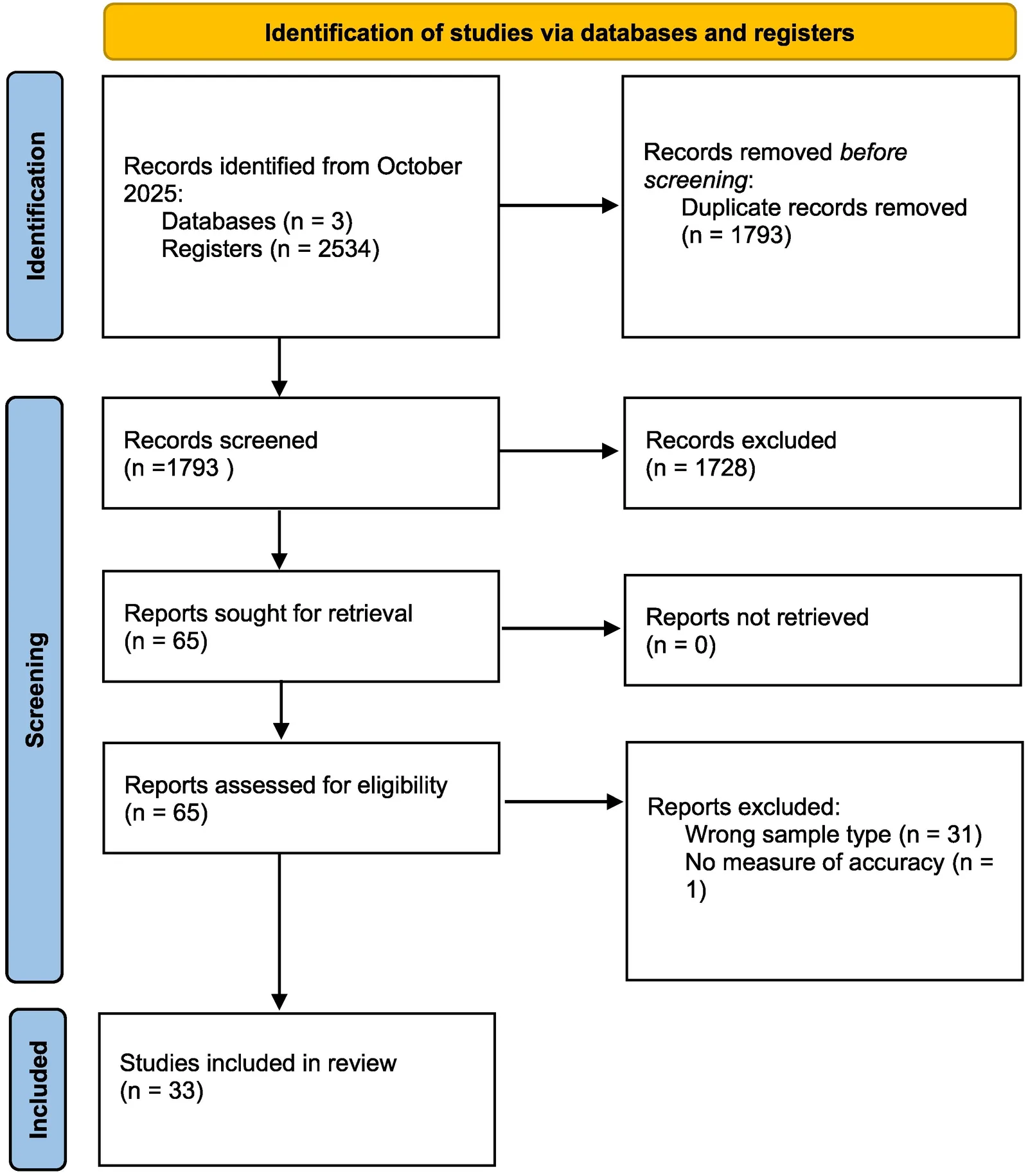
Identification of studies included in the review.

Article titles were uploaded to the rayyan.ai screening tool^15^ where the results were de-duplicated before manual screening. Two review authors (S.H.N. and A.A.) performed the primary screening by title and abstract according to the inclusion criteria and the full text articles were retrieved. We included all records that indicated a comparison of a new index method to a comparator that could be associated with urine samples, direct sampling and UTIs. Disagreement in selection was resolved through discussion. Following initial screening, 65 full text articles were screened. Secondary screening of the full text articles was conducted by three reviewers (S.H.N., R.S. and A.A.). Disagreements were resolved by discussion and reasons for 31 exclusions are shown in Figure 1.

### Data extraction and management

Three review authors (S.H.N., R.S. and A.A.) extracted data using a data extraction form for characteristics indicated in the headings within Table 2. Any disagreements were resolved through discussion and cross-checking data. For studies that did not report an overall categorical agreement (OCA), these were calculated on the basis of the summary data tables reported in that study.

**Table 2.** Summary of findings

Author	Year	Country	Assay duration	Population type	Sample (*n*)	Antibiotics used (*n*)	Organisms included	Sample pre-treatment	Index method	Reference method	Resistance prevalence reported?	Presence of bacteriostatic?	CA (%)	Reference
Index method: disc diffusion														
Mukerjee and Reiss-Levy	1994	Australia	< 24 h	MSU and CAUTI	324	6	Gram-negative monomicrobial samples with ≥10^9^ CFU/L by microscopy.	Diluted 1:100 in saline	Direct disc diffusion	Disc Diffusion	Yes	Unclear	98.5	^16^
Breteler *et al.*	2011	The Netherlands	16–20 h	Microbiology laboratory urine. Hospitalized patients	100	12	Single organism Gram-negative samples.	None	Direct disc diffusion	BD Pheonix. Etest used for discrepant results	No	Unclear	96.01	^17^
Périllaud-Dubois *et al.*	2018	France	8 h	Microbiology laboratory urine	193	22	Gram-negative monomicrobial culture with >5×10^4^/mL leukocyturia.	None	Direct disc diffusion	Disc diffusion	No	Unclear	97.9	^18^
Johnson *et al.*	1995	USA	16–18 h	Women with acute cystitis taken using a midstream clean-catch sample. Acute cystitis defined by ≥8 leukocytes mm^3^, dysuria and pyuria	129	25	All samples including Gram-negative and Gram-positive and mono and polymicrobial cultures (no more than 2 different Gram-positive bacteria).	None	Direct disc diffusion	Disc diffusion	Yes	Unclear	95.5	^19^
Sundqvist *et al.*	2014	Sweden	16–20 h	Microbiology laboratory urine	188	5	Enterobacterales with >10^4^ CFU/mL.	None	Direct Disc Diffusion	Disc diffusion	Yes	Yes	98.2	^20^
Mohammad and Omer	2018	Iraq	18–24 h	Microbiology laboratory urine	206	10	Gram-negative and Gram-positive monomicrobial cultures.	None	Direct disc diffusion	Disc diffusion	Yes	Unclear	97.1	^21^
Zboromyrska *et al.*	2016	Spain	18–24 h	Hospital recruitment	83	Unknown	Gram-negative and Gram-positive monomicrobial cultures.	Pelleted and resuspended to 0.5 McFarland	Direct disc diffusion	Disc diffusion	No	No	100	^22^
Dornbusch *et al.*	1979	Sweden	18 h	GP recruitment	156	4	Gram-negative and Gram-positive monomicrobial and polymicrobial cultures.	None	Dipslide direct disc diffusion	Disc diffusion	Yes	Unclear	91.7	^23^
Oakes *et al.*	1994	Australia	18–24 h	Microbiology laboratory urine, MSU and Catheterized samples	1106	6	Gram-negative and Gram-positive monomicrobial cultures.	None	Direct disc diffusion	Disc diffusion	No	Unclear	94.3	^24^
Kannan *et al.*	2015	India	16–18 h	Suspected UTI, MSU	62	7	Gram-negative and Gram-positive monomicrobial cultures.	None	Direct Disc diffusion	Disc diffusion	Yes	No	100	^25^
Scully *et al.*	1990	Ireland	16–18 h	General Practice	58	3	Gram-negative and Gram-positive monomicrobial cultures.	None	Direct Disc diffusion	Disc diffusion	Yes	Unclear	94.5	^26^
Kjaerulff	1986	Denmark	18–24 h	General Practice	232	4	Gram-negative and Gram-positive monomicrobial cultures and polymicrobial cultures.	None	Dipslide direct disc diffusion (Sensicult)	Disc diffusion	Yes	Unclear	78.7	^27^
			18–24 h	General Practice	193	6	Gram-negative and Gram-positive monomicrobial cultures and polymicrobial cultures.	None	Direct Disc diffusion (Bact-plate)	Disc diffusion	Yes	Unclear	84.2	
Jain and Nikita	2023	India	16–18 h	Outpatients or hospital patients with suspected UTI	58	14	Gram-negative and Gram-positive monomicrobial cultures.	None	Direct Disc diffusion using Chromogenic Agar	Disc diffusion	Yes	Unclear	86.2	^28^
Mugiraneza *et al.*	2020	Korea	4–8 h	Microbiology laboratory urine	103	10	Enterobacterales monomicrobial cultures ≥2×104 CFU/mL.	None	Direct Disc diffusion	Vitek 2	No	Unclear	93.3	^29^
Ilki *et al.*	2020	Turkey	18–24 h	Microbiology laboratory urine	111	14	Enterobacterales monomicrobial.	Pelleted, washed, resuspended to 0.5 McFarland standard	Direct Disc diffusion	Disc diffusion	No	Unclear	95.5	^30^
Hollick and Washington II	1976	USA	16–18 h	MSU	246	8	Gram-negative and Gram-positive monomicrobial cultures and polymicrobial samples.	None	Direct Disc diffusion	Disc diffusion	No	Unclear	95.5	^31^
Angaali *et al.*	2018	India	<24 h	De-identified microbiology laboratory urine with turbidity >0.5 McFarland	46	17	Gram-negative monomicrobial samples.	Centrifuged and diluted to 0.5 McFarland turbidity in saline	Vitek 2	Vitek 2	No	Unclear	94.3	^32^
Torres-Sangiao	2022	Spain	7–18 h	De-identified microbiology laboratory urine. Screened for positive bacteriuria	211	16	Single organism, *E. coli* with >150 bacteria/mL.	None	Direct Vitek2	Vitek2	No	Unclear	97.4	^33^
Bongard *et al.*	2015	UK	16–18 h	Microbiology laboratory urine	40	5	Unknown.	None	Flexicult SSI urinary kit	Disc diffusion	Yes	Unclear	83	^34^
Index method: particle imaging, counting and microscopic capture														
Zhang *et al.*	2021	USA	90 minutes	De-identified microbiology laboratory urine	55	1	Single organism (>98% Gram-negative).	Warmed to 37 C for 30 minutes, Filter through 5-µm syringe filter and diluted at least 1:10 in Luria–Bertani (LB) medium	Particle imaging	BD Pheonix	Yes	Unclear	98	^35^
Alonso-Tarrés *et al.*	2024	Spain	45 minutes	Women, uncomplicated UTI symptoms <7 days, no antibiotic treatment, not pregnant, >18 years old, MSU	89	5	Culture positive for *E. coli*, *K. pneumoniae*, *P. mirabilis*, *S. saprophyticus* and/or *E. faecalis*.	None: Urine added to cartridge which is automatically filtered in the machine and MHBII is added	PA-100 AST (Phase contrast microscopy)	Agar Dilution (Fosfomycin) and Disc diffusion. Broth microdilution was used for discrepant results	Yes	No	92.1	^36^
Toosky *et al.*	2019	Italy	2 h	Microbiology laboratory urine from both in and outpatients	25	5	Gram-negative and Gram-positive and mixed organism samples.	Diluted in LB broth to a density of ∼5×10^4^ CFU/mL	Particle imaging	Vitek2	No	No	90.7	^37^
Burg *et al.*	2022	USA	6 h	Microbiology laboratory urine	132	4	*E. coli*	Urine is processed through a size exclusion column to isolate bacteria, remove urine and boric acid. Incubated with MHBII and antibiotics for 4 h before addition of FISH reagents	Counting of FISH labelled bacteria bound to magnetic particles	Broth Microdilution and or disc diffusion	Yes	Yes	92.3	^38^
Zhang *et al.*	2020	USA	1 h	De-identified microbiology laboratory urine	30	2	Gram-negative and Gram-positive monomicrobial cultures.	Warmed to 37 C for 30 min and passed through a 5-μm filter to remove large substances and diluted 1:10 in LB	Video of individual cells	BD Phoenix	Yes	Unclear	100	^39^
Gilboe *et al.*	2021	Norway	24 h	Microbiology laboratory urine from outpatients including GP and nursing homes	56	10	Gram-negative and Gram-positive monomicrobial cultures.	None	Flow cytometry	MicroScan for *E. coli* and EUCAST methods for other organisms	No	Yes	99.1	^40^
Nicolai *et al.*	2021	Italy	3 h	Inpatient and outpatient	87	6	Single organism, Gram-negative and Gram-positive.	Diluted in LB broth to a density of ∼5×10^4^ CFU/mL incubated for 3 h at 37°C before diluting 1:100 in SYTOX Orange and heating at 80°C for 7 min	Fluorescence particle counting	Vitek2	Yes	Unclear	81.2	^41^
Index method: mass spectrometry, nucleic acid quantification, growth in broth/agar														
Neuenschwander *et al.*	2023	Germany	4.5 h	Hospital inpatients including catheter samples	105	6	Gram-negative, specifically Enterobacterales.	Pelleted, washed, resuspended, medium and diluted to 0.1 OD_600_. Incubated for 2.3 h at 37°C and lysed and antibiotic spectra measured	MALDI-TOF MS method following incubation with antibiotics	MIC Etest	No	Unclear	94.7	^42^
Kapor and Gupta	2017	India	4 h	Microbiology laboratory urine	243	14	Unknown.	Urine sample is filtered and resuspended in media and added to wells containing antibiotics	Chromogenic endpoints	Disc diffusion	No	Unclear	92.6	^43^
Behere and Haldar	2024	India	6–9 h	De-identified microbiology laboratory urine	50	4	Gram-negative monomicrobial cultures.	Diluted in antibiotics and incubated prior to testing on redox dye containing membrane	Redox dye colour change	Laboratory gold standard	Yes	Unclear	100	^44^
Chen *et al.*	2022	USA	3.5 h	Microbiology laboratory urine	9	3	Enterobacterales and *Pseudomonas aeruginosa*.	Pelleted and resuspended to 0.5 McFarland	Quantification of rRNA following 3 h incubation with antibiotics	Vitek and Broth Microdilution	Yes	Unclear	100	^45^
Heinze *et al.*	1979	USA	4–6 h	Inpatients and outpatients	86	10	Gram-negative monomicrobial cultures.	Diluted to approximately 10^7^ CFU/mL based on Gram-staining counts	Turbidity in the presence of antimicrobial elution discs	Disc diffusion	No	Unclear	93	^46^
Brönnestam	1999	Sweden	18 h	Microbiology laboratory urine	882	8	Single organism samples, excluding Staphylococci and Streptococci.	None	Direct Agar Dilution using Mast Multipoint Inoculator	Disc diffusion	No	Unclear	95.8	^47^

### Risk of bias of included studies

An initial quality assessment was completed using the QUADAS-2 tool.^48^ Each domain was assessed for the risk of bias and was performed by reviewer S.H.N. In this scoping review, the following four domains were assessed for risk of bias: patient selection, index test, reference standard, and flow and timing.

### Understanding the effect of lower resistance prevalence on confidence intervals for sensitivity

A set of theoretical data tables were created with the following assumptions. True resistant, true susceptible, false resistant and false susceptible sample numbers were simulated for an index test performing with 95% sensitivity versus a reference test. Data tables were created covering sample sizes ranging from 100 to 3200 with a prevalence of resistance of 50%, 10% or 5%. For each simulated data table upper and lower confidence intervals at a 95% level were calculated for sensitivity using the Klopper–Pearson method, and these confidence intervals were plotted against sample size.

## Results

### Study selection

The literature searches (conducted October 2024) identified 2534 publications since 1976. After removal of duplicates and screening against titles and abstracts, this resulted in 63 articles that were assessed against the inclusion criteria, which delivered 33 studies (Figure 1). The details of these 33 studies are outlined in Table 2.

Most search results that were not included were excluded because they failed to meet the inclusion criteria due to not testing the correct sample type. For example, one study that reports the use and quality of point-of-care urine AST in general practice was not included, because of the sample type studies. Although this particular study reported an evaluation of several relevant direct-from-urine methods—including Flexicult SSI urinary kit and a Mueller–Hinton agar method—only simulated urine samples containing a standardized uropathogenic bacteria were tested.^49^ This study therefore did not meet our inclusion criteria of testing urine samples from patients with suspected or confirmed UTI.

### Study characteristics

A total of 33 studies met the inclusion criteria. Nearly half of all studies included (44%) were published since 2015, indicating an active current interest in direct-from-urine testing and novel methods to achieve faster AST results for patients with suspected UTI.

Most of the studies tested urine samples from the microbiology laboratory (20/33). For most of these, the age, patient population and original source of the urine samples (e.g. primary versus secondary care) were unknown or not reported.

The most common index test used was direct disc diffusion (19/33), in which the urine sample was streaked onto a Mueller–Hinton agar plate, antibiotic discs were added to this plate and zones of inhibition around the disc were examined manually after overnight incubation. From our searches, we found that the first report of direct-from-urine disc diffusion was published in 1976.^31^ A total of 19 studies assessed variations of this approach. Sample sizes in these studies ranged from 58 to 1106. The largest study, comprising 1106 samples,^24^ assessed the diverse range of sample types arriving to a laboratory (e.g. outpatient midstream urine plus inpatient catheterized), testing susceptibility against six antibiotics. For most of these studies, the index test was urine plated directly on a disc-diffusion plate without additional sample pre-treatment or processing. The conventional two-stage AST method—involving picking isolates from overnight urine plating followed by disc-diffusion testing of these isolates—was the reference method for all these studies.

Most studies (27/33) only reported the CA results analysed for the subset of urine samples in which a single organism was detected. In two studies, the composition of bacteria found in the samples was unclear or not stated. Five studies did include results from samples with two or more organisms present; however, the method used for data analysis in these studies was not clearly stated. This may be because a sample containing two or more organisms cannot be directly scored as susceptibility or resistant; instead, the susceptibility of each organism isolated may have been tested separately by the reference test, leading to ambiguity in how agreement with the index test would be classified and therefore how CA would be calculated.

We assessed the different types of analytical method used in the included articles. We identified a range of methods for performing AST directly from urine samples that used a variety of distinct sample processing methods, and examples of departure from current reference methods. A proportion of these studies incorporated adjustments to reference AST methods with the aim to standardize the bacterial inoculum within the urine sample by dilution and/or centrifugation and at the same time avoid any risk of urine matrix components interfering with the AST method. All but one study required at least one additional manual step. Adjustments included standardization of the inoculum, including adjusting the density and purity of the organisms (e.g. by centrifugation and removal of urine supernatant); adjusting the matrix (e.g. by resuspending a centrifuged bacterial pellet in Mueller–Hinton broth) and modifying incubation time before determining the results. One study used a proprietary device that featured full automation, from adding a patient urine sample to the device to outputting an antibiotic susceptibility result; by contrast, other studies reported methods with multiple steps performed by laboratory staff.

Direct-from-urine testing studies were performed with three commercial platforms: bioMérieux Vitek 2, Flexicult SSI kits and the Sysmex PA-100 AST system. The Vitek 2 is not indicated for direct-from-urine testing in the instructions for use. Direct urine analysis by Vitek 2 was compared to the Vitek 2 system operated under normal conditions testing isolates from overnight culture, reporting a CA of 94.3%.^33^ Likewise, the direct agar dilution of SSI Flexicult was compared to reference disc diffusion of isolates, reporting CA of 83%.^34^ The Sysmex PA-100 AST system is indicated for use with direct testing of urine samples, and in this study was found to have an OCA of 93.1% compared to reference disc diffusion.^36^

### Risk of bias

**Figure 2.**
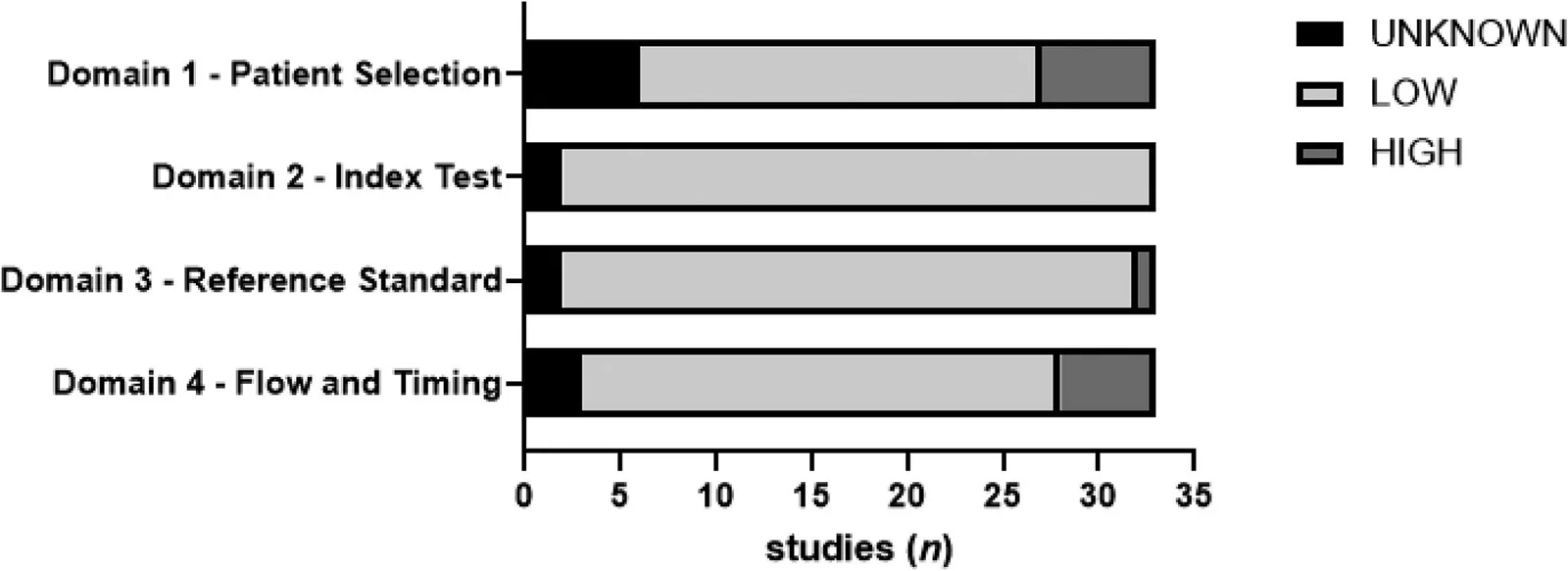
Risk of bias quality assessment of included studies. Distribution of quality assessment scores using QUADAS-2 tool across the 33 included studies.

An initial quality assessment was performed using the QUADAS-2 tool assessing the patient selection, index tests, reference standard used, and the flow and timing of the study. Figure 2 summarizes bias scores for the included studies across these four domains. It was not intended to fully capture all aspects of quality assessment of diagnostic accuracy described by QUADAS-2 studies. For example, it proved difficult to systematically assess the quality of reported analytical performance, as discussed below. However, we were able to assess risk of bias within this scoping review.

Most studies used leftover urine from samples already being tested at microbiology laboratories (diagnostic remnants). These samples have the potential to represent a broad diversity of patient demographics but in many cases the demographics of samples used were unknown. Unlike other diagnostic tests, AST is not used for diagnosing a disease. The resistance rates for different antibiotics will vary by geographic location and changes over time. The distribution of resistance rates may affect the confidence in the accuracy of these studies if they have not been considered. This was not assessed as part of the risk of bias but is explored as a limitation of the studies. However, since these studies were focused on the accuracy of AST methods, this was classified as low risk of bias here.

The reference standard was classified as low risk if a known validated method or commercial test was used, for example, disc diffusion or Vitek 2. As recognized methods they are assumed to give an accurate result. The clinical reference standard method for most antibiotics and species is broth microdilution, but this is not always used in clinical settings where disc diffusion is more common as it requires less labour, space and consumable resources. All current reference methods require urine plating followed by selecting isolates for subsequent AST.

The index test was classified as low risk if the method was presented in a replicable manner and details were provided about how the data was analysed: for example, if the method was pre-specified or if they were performed without knowledge of the reference test result. For some of the more novel methods reported, the study was classified with a higher risk because the analysis is described but it was unclear whether the method was pre-specified and fully blinded from the reference standard.

In an ideal design, the reference standard would be broth microdilution, tested at as close a time as possible to the method being evaluated.^50^ However, as the index tests are used directly on urine samples, there is an unavoidable delay between performing the index test versus the reference standard. In most tests, the pre-specified thresholds were used, i.e. disc-diffusion interpretation and automated analysis methods for Vitek 2.

The areas that had the highest degree of potential bias were patient selection and flow and timing. Patient selection was due to exclusion of samples for analysis or restricted patient recruitment that would affect applicability, such as restricting analysis of samples to only *Enterobacterales.* Whereas most studies excluded the analysis of polymicrobial or mixed samples, this was not classified as high risk as the clinical importance of mixed samples is not clear and current methods often do not analyse mixed samples. However, further investigation may be warranted to better understand how to interpret test results where multiple organisms are identified. While many samples were collected from hospital microbiology laboratories, it was often unclear whether these were collected randomly. For flow and timing, some studies were unclear on whether researchers had access to reference information before analysis and the reasons for excluding some samples/patients from analysis were unclear. Most studies provided enough methodological detail for the reference and index tests used to be classified as low risk.

## Primary outcome

### Accuracy of direct-from-urine AST

**Figure 3.**
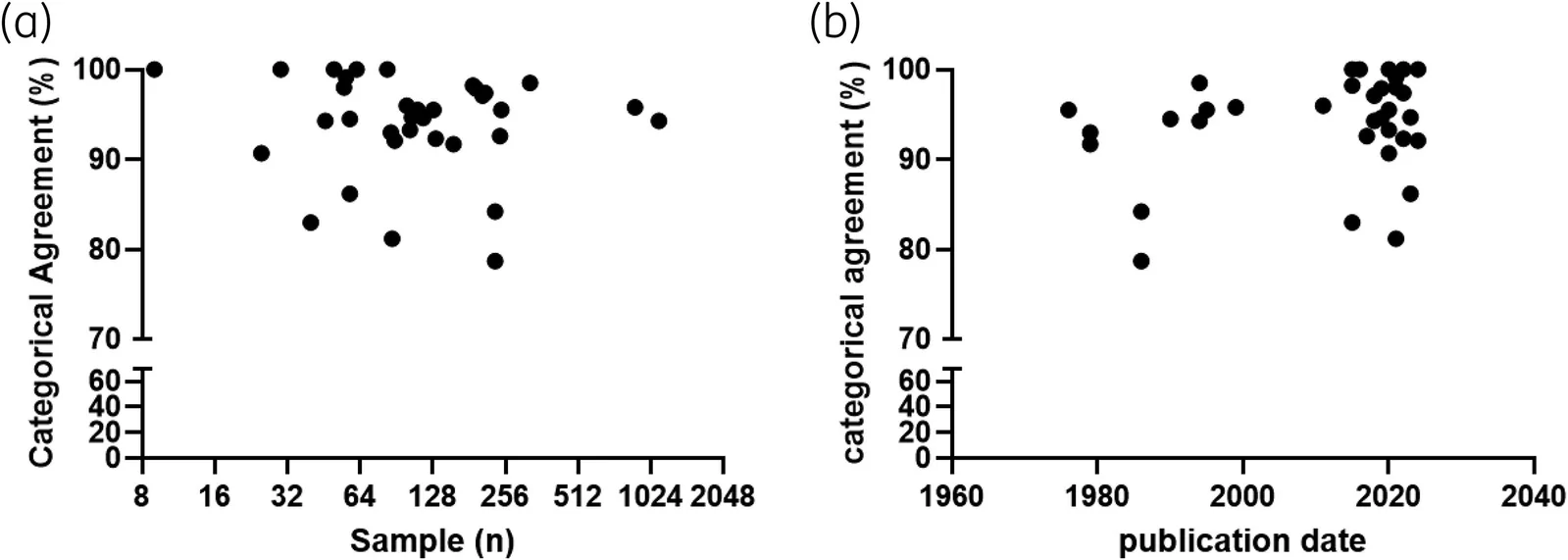
Summary of CA and key characteristics of direct-from-urine AST. (a) CA against sample size. (b) CA against publication date.

Categorical agreement (CA) was the most common measure of accuracy reported, which indicates the concordance between a direct AST result and a reference method. The studies included from this initial selection reported a wide range of CA from as low as 78.7% for the study reporting the lowest accuracy, up to complete agreement (i.e. 100% CA) between the index test and the reference test for the study reporting highest accuracy. The distribution of reported CAs reported across all studies is illustrated in Figure 3. However, many of the studies had low sample numbers and either a low distribution of susceptible to resistant organisms, or this distribution was not reported, therefore the confidence limits for the reported CA can be expected to vary significantly between these studies. Furthermore, the level of agreement, and especially the confidence limits of estimation of other accuracy measures such as sensitivity and specificity, may also differ by antibiotic.

Studies which reported CA for Gram-negative or Gram-positive monocultures using a direct disc-diffusion method were the most common and we suggest that there is now a growing and significant accumulation of data to support the accuracy of results reported for direct-from-urine disc diffusion with these samples. Accuracy ranged from 93.3% to 100%^21,28,44^ with a sample size ranging from 58 to 1106 samples for these studies.^23,39^

Overall, only four studies, using five different tests, reported CA that fell below the 90% that is suggested as a minimum accuracy in the World Health Organization (WHO) Target Product Profile for rapid AST methods.^26,27,31,33^ Of these, two studies included polymicrobial samples in their calculated accuracy;^26,27^ for one study it was unclear whether polymicrobial samples were included or not;^31^ and in one study a chromogenic media other than Mueller–Hinton Agar was used for direct urine disc diffusion.^27^ It has previously been observed that media formulations other than Mueller–Hinton Agar can affect AST results,^51^ potentially explaining this apparently lower accuracy in this study.

The method in which the largest number of publications reported CA at or above 95% was direct-from-urine disc diffusion, but this was also the most frequently studied method for rapid direct AST so, although the body of evidence is greater for this method, we cannot conclude this is a more accurate method than other methods that have been assessed in fewer studies. Sample sizes for most of these studies were above 100, and the number of antibiotics tested ranged from five to 25. All 17 of these studies reported CA >95% for monomicrobial samples. Some direct disc-diffusion-based analyses reported 100% concordance with a reference method that was also disc diffusion.^22,25^ The study by Zboromyrska *et al.*^22^ reported that when samples with monomicrobial infections were considered all results were concordant, whereas for polymicrobial samples a separate analysis was explored. The study by Paul *et al.*^25^ determined direct AST separately for Gram-positive (13 isolates) and Gram-negative (49 isolates) monomicrobial cultures. With a high rate of resistance in Gram-negative organisms, the CA remained at 100%, albeit with a low sample size that reduces confidence in this group.

An interesting method was used by Zhang *et al*.^35^ in which cell division of individual cells was tracked by imaging light scattering by bacterial cells using digital video. Considering ciprofloxacin for treatment of 30 clinically positive UTI samples, they demonstrated gradually improving CA with increasing incubation time before analysis (87% at 30 minutes, 94% at 45 minutes and 100% at 60 minutes) compared with reference testing by the BD Phoenix AST system. While this study reports useful information about novel method optimization, the sample size is too small to draw meaningful conclusions about the overall accuracy of this approach, and this particular study should be considered a technological proof-of-concept rather than validating clinical accuracy.

A small subset of studies that observed higher (>99% CA) or lower (<90% CA) overall agreement were individually examined to understand whether differences between these studies could explain the range of reported accuracy. For one study that reported an overall CA of 82.3%, the lower accuracy may potentially be attributed to inclusion of fosfomycin, as the sensitivity predicted value was found to be 86.7% for this antibiotic, in contrast to five other antibiotics for which the sensitivity predicted value ranged from 93.9% to 100%.^41^ In this study, a novel particle counting method was evaluated with inpatient and outpatient samples. For mixed samples in this study, hospitals did not perform AST and no antibiotic was indicated, so the accuracy for this subset of samples could not be calculated. It is unclear whether the lower agreement for fosfomycin reflects the performance of the index direct testing method, or higher variation in AST determination by the reference method that can occur for this antibiotic.

The number of studies reporting direct-from-urine AST have accelerated in the last 10 years. Nearly half of all studies included (44%) were published since 2015 (Figure 3b), indicating an active and current interest in direct and novel AST methods for UTI. However, the inclusion of studies as far back as 1976 leads to temporal heterogeneity that affects the feasibility of meta-analysis. A proportion of the included studies are relatively old (19 of 33 published within the last 10 years, only 10 within the last 5 years), and during the past decade the available methods and technologies in this space may have evolved. Furthermore, the profile of organisms, distribution of minimum inhibitory concentrations (MIC), antibiotic breakpoint and prevalence of resistance may have changed over this period. This could further limit the validity of like-for-like comparisons.

## Secondary outcomes

### Study size and design

**Figure 4.**
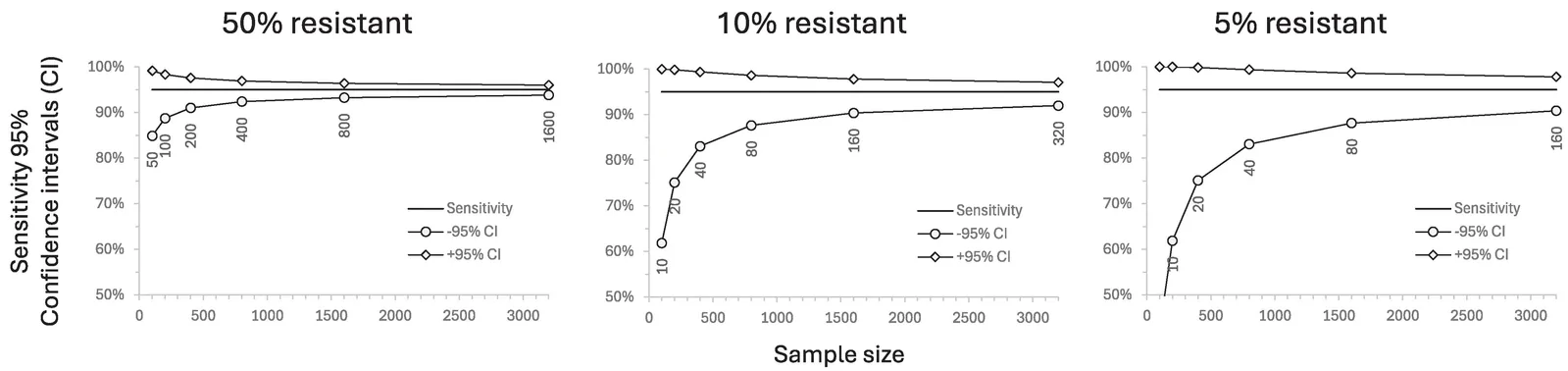
Illustration of the effect of prevalence of resistance on confidence in calculated sensitivity to detect resistance. Sets of simulated data for a test with 95% sensitivity were created where 50%, 10% or 5% of the samples were positive for resistance, and 95% confidence intervals (CI) for sensitivity calculated with the Klopper–Pearson method. These ranges were plotted against overall sample size for each simulated population, with the number of resistant organisms indicated as data labels.

The studies ranged from smaller proof-of-concept evaluations of new methods or technologies to large scale evaluations of methods or commercial products. The number of urine samples assessed by each method ranged from nine to 1106 samples, and from one to 25 different antibiotics were assessed. The sample size required to confidently report accuracy will depend on the prevalence of resistance in the population tested. Surveillance data for many of the antibiotics assessed, and the years the studies were undertaken, indicates that the prevalence of resistance could be expected to range from 0% up to 50%.^52^ On the basis of an expected sensitivity and specificity of 90% and a precision of 0.05 and confidence of 95%, a sample size of 277 samples would be appropriate to confidently report accuracy for samples containing around 50% resistance rates. However, for antibiotics with a low prevalence of resistance (∼10%), such as nitrofurantoin, a sample size of >1000 would be required. Whereas some studies published the prevalence of resistance, others simply stated the CA, indicating a possible bias in resistance profiles of urine samples tested. The impact of prevalence of resistance on 95% confidence intervals for the sensitivity of detecting resistance was illustrated in Figure 4. It can be concluded that only the largest of studies identified here are sufficiently powered to confidently indicate sensitivity of resistance detection for antibiotics with the lowest resistance rates. Notably, the large sample size of 1106 reported by Oakes^24^ should provide more confidence in sensitivity of resistance detection for low prevalence resistances. Oakes *et al.* used a direct disc-diffusion method and attained 94.3% CA with monomicrobial cultures, including samples containing Gram-positive and -negative organisms. Although this larger size should capture the required number of resistant isolates for antibiotics with low prevalence, the prevalence of resistance was not reported for the antibiotics tested within this study.

Gram-negative bacteria are the predominant causative pathogens for both complicated and uncomplicated UTIs.^53^ A total of 14 studies limited sample inclusion to only assess or present data from a Gram-negative single organism containing samples. In some studies, these were further limited to *Enterobacterales* or *Escherichia coli* only. The findings of these studies should be qualified by an understanding of this limitation of focussing on only the most commonly identified class of uropathogens.

### Time to result

**Figure 5.**
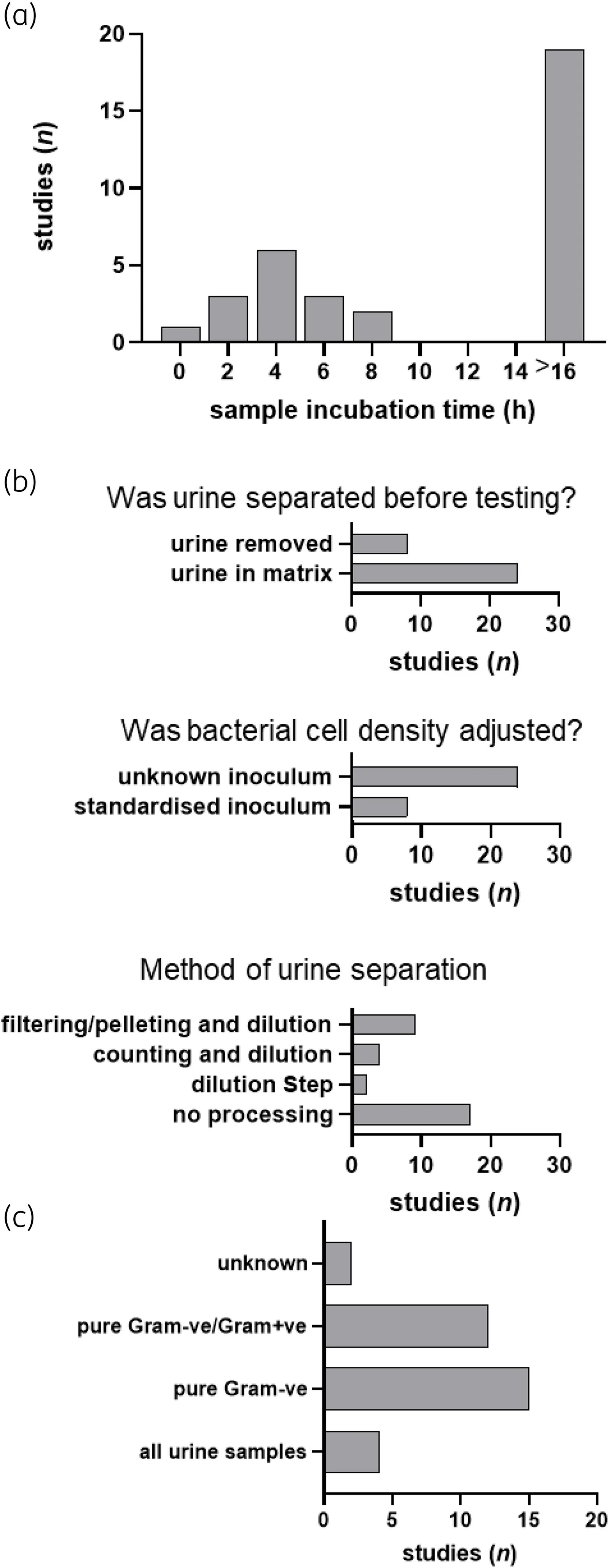
Characteristics of direct-from-urine AST methods evaluated in included studies and reported method to assess accuracy. (a) Plot illustrating distribution of incubation times used for AST step. The ‘>16 h’ indicates all studies that included overnight incubation. (b) Illustration of steps used by AST method to adjust bacterial cell density and/or remove interfering urine matrix components before susceptibility testing. (c) Summary of sample types included in accuracy calculation.

All reference methods required overnight incubation for isolation of a pure culture and for 18 studies the reference AST method required a second subsequent overnight incubation. Thus, all the direct-from-urine index tests offer the potential to cut the time to result by at least one overnight incubation step by avoiding isolation of pure cultures before AST. The range of assay times in the studies identified is illustrated in Figure 5A. Other studies have reduced the overall method time further, with the shortest time to result reported at 45 minutes.^36^ Most studies reported an assay time of around 4 h, offering the potential for same-day AST results.

Particle imaging, microscopic analysis and counting, and videography of individual cells were comparatively quicker methods, requiring 45 minutes to 6 hours. All except one of these methods required a sample pre-treatment step—such as filtering, dilution and/or columns—to remove any potential interfering substances or particles, or to separate bacterial cells from other urine matrix components. The analysis of these studies included samples containing single organism cultures including Gram-positive and -negative as well as mixed culture samples. Time to result is likely to be fastest when testing rapidly dividing Gram-negative bacteria such as *E. coli*, whereas other organisms may have longer doubling times such as Enterococci. Turbidity based methods are also influenced by different organisms’ size and shape, such as *E. coli* which is typically larger than Staphylococci, altering the intensity of turbidity for the same bacterial cell concentration.

All studies described the analytical assay time, but none of the studies reported overall time from sample collection through to results reporting. It should be noted therefore that in a routine diagnostic microbiology laboratory, the duration of the analytical method represents just one aspect of reporting, and slower reporting times can arise due to additional logistical, pre-analytical and reporting steps.

### Processing steps used to separate bacteria from urine prior to direct-from-urine AST

Most studies identified assessed a direct disc-diffusion method without pre-processing of the sample, where urine samples were streaked onto agar without dilution and antibiotic discs directly added to the plate. This method has the benefit of simplicity; however, it can be prone to low inoculum numbers, in some cases growth being too low to clearly read a zone of inhibition. Urine sample matrix components, depending on exact methodology and any sample dilution steps, also have the potential to interfere with microbial growth and response to antibiotics, which can affect accuracy.^54^ The distribution of studies across distinct urine processing methods is illustrated in Figure 5.

For these reasons several studies report methods that incorporate significant pre-treatment steps to remove urine matrix components—or any other substances in the urine such as bacteriostatic boric acid added to the urine sample—that could affect microbial growth. In some cases, this was followed by resuspension of any bacteria in the urine to an estimated inoculum. This is intended to address two concerns about direct-from-urine testing, the effect of sample matrix interference (e.g. urine components affecting response to antibiotic) and variation in inoculum density.

Five studies included pelleting the urine by centrifugation and resuspending at a specific optical density^22,30,42,45,55^. For other methods, the urine samples were filtered. In two studies, this was to remove large debris such as blood cells or casts from the samples. The filtered urine was further processed by dilution; thus urine matrix would still be present in the final sample.^35,39^ In two other studies, filtering was used to remove the urine matrix altogether and recover bacterial cells that were subsequently resuspended in media.^38,43^

Of note, one method had a fully automated approach that did not require the user to process the urine sample, but the urine matrix was removed from the sample during testing.^36^ This nanofluidic system captured the bacteria while removing the urine and exposed the bacteria to different antibiotics.

## Discussion

### Accumulating evidence of the accuracy of direct-from-urine AST

Many of the studies presented here contribute to a significant accumulating evidence base establishing the accuracy of the direct-from-urine methods for the determination of antibiotic susceptibility, indicating that CA of >90% can be achievable for the most commonly presenting sample type for UTI: a single organism Gram-negative infection. This level of agreement is generally considered acceptable^56^ and meets the WHO Target Product Profile for AST. Even studies that use a simple modified disc diffusion for direct-from-urine sample testing have the potential to provide benefits in time to result of at least one overnight incubation step over current reference standard methods. A diverse range of direct sample testing approaches for urine have been reported, using different approaches ranging from adaptations of disc diffusion, to integrated technology such as microfluidics and miniaturization of culture,^57^ single particle/cell tracking^39^ and single cell droplet AST.^58^ New commercial tests can fully automate the AST process, such as the Sysmex PA-100 System that reports phenotypic AST results from specimen within 45 minutes for five antibiotics.^36^

There are clear accuracy targets that will need to be met for novel AST systems to be used in routine clinical microbiology. According to ISO 20776-2, qualitative AST devices or methods should achieve >95% sensitivity and specificity when compared with a reference method; quantitative MIC measurements should likewise achieve >90% essential agreement with <±30% bias in MIC values.^2^ However, it is also well documented that there can be significant variation in current reference AST methods results within and between laboratories. One example study assessed the CA of AST for 12 challenge isolates comparing nine laboratories using disc-diffusion and commercial AST systems, and found categorical agreement varying across 72.3%–100% depending on the method, organism and/or antibiotic used.^59^ The accuracy of novel AST systems should therefore be judged in the context of this variability in results from imperfect reference method.

### Technical challenges for direct-from-urine AST

There are several potential technical challenges that direct-from-urine testing must overcome. Four proposed challenges are: (i) unknown pathogen; (ii) unknown inoculum cell density; (iii) matrix interference and (iv) samples containing polymicrobial populations.^60,61^ Reference methods test a pure culture, with known identity, suspended at a defined inoculum, and assess the impact of antibiotic on growth in a defined medium at concentrations selected on the basis of agreed breakpoints to determine the susceptibility or resistance for a given organism (ISO 20776-1;2019). First, the conditions used in reference AST methods—most notably the breakpoint antibiotic concentrations—vary between species, thus reference methods typically require identification before AST to select the correct antibiotic concentration for that species. Second, bacterial cell density in a patient urine sample varies over a high dynamic range, from >10^8^ CFU/mL down to 10^2^ CFU/mL, dependent on population group.^1^ Third, urine matrix components can interfere with bacterial response to antibiotics, potentially affecting AST results. Urine contains various components that might alter bacterial growth during AST, such as variations in pH, presence of antimicrobials or host factors.^54^ Finally, samples may contain more than one organism,^62^ while the clinical relevance of polymicrobial samples remains debated, there are no reference methods for interpreting an AST performed on polymicrobial populations. However, while there is evidence to show that these limitations are known to affect AST results for specific urine compositions or bacterial strains, there has been no review to date that has examined the breadth of studies that report accuracy of direct-from-urine AST methods.

The exact method used to perform direct-from-urine AST would be expected to affect the impact of these various potential interfering factors on accuracy. Urine can interfere with broth microdilution by changing the pH of the media, and this effect can vary for different antibiotics.^54^ This urine matrix effect may be less likely to affect disc diffusion, as the urine is diluted into the larger media volume of the agar plate (10–15 mL per agar plate compared to 0.1 mL in broth microdilution wells) and interfering substances may diffuse away. This is true for other interfering factors which might be present in the urine samples, such as metabolites and glucose levels, Vitamin C, transport media or medications (such as antibiotics) excreted via the urine.

### Study design

Studies designed to evaluate direct-from-urine AST methods or devices using urine samples submitted to the routine diagnostic laboratories have the benefit of including a wide diversity of patient samples. In these studies, the samples are typically collected randomly, and thus additional data may not be available, such as the sex, age, previous test results or other demographic indicators and sample characteristics of different patient groups. This approach allows the index method to be tested using a population that is representative of that diagnostic service, and with samples that would be typically seen within a clinical laboratory. This may make the accuracy assessment more relevant to routine testing practices in which patient information for each urine sample is frequently unknown at the point of testing and reporting. Alternatively, some studies were designed to focus on a specific patient group, such as women aged between 18 and 35 years old presenting with an uncomplicated UTI. These more focused studies may be more appropriate for specific use cases such as community or primary care testing pathways.

Approximately half of these studies included here (15/33) could be classified as proof-of-concept reports rather than full evaluation studies. This is due to smaller sample sizes and a focus on developing new technologies or method rather than testing a commercially available technology or established AST protocol. Furthermore, studies investigating new technologies may be subject to positive result bias. There is a potential for conflict of interest, particularly in cases where the technologies under evaluation are in early stages of development and may be commercially viable in the future. In such instances, only favourable outcomes may be selectively reported, which could distort the overall conclusions of this scoping review.

### Remaining evidence gaps and future perspective

There is significant potential for faster methods to test urines—especially novel rapid AST tools—to improve treatment and/or reduce antibiotic misuse, and to thereby avoid ineffective treatment with empirically selected antibiotics. This urgent clinical and public health need for faster, effective microbiology testing continues to drive research into rapid AST methods and technology. However, it should be noted that professional bodies such as EUCAST do not currently recommend direct-from-urine methods.^63^ In spite of promising reports of the accuracy for direct-from-urine testing across many individual studies, there remain gaps in the current evidence requiring further validation studies for the use of direct-from-urine AST methods in clinical workflows. This scoping review strongly suggests that improved standardization of studies that evaluate direct-from-sample AST methods would be helpful to improve rigour and robustness of the evidence base, and make it possible to systematically compare conclusions across studies. Key information should be reported including prevalence of resistance and calculation of confidence limits for key metrics, and more transparency is needed in presenting data that fill current gaps in evidence, such as reporting results for samples containing more than one organism.

For many studies, accuracy was calculated by analysing data from samples with a single organism present (determined by plate culture). For 14 studies, only samples containing a single Gram-negative species were considered. However, this narrows the type of sample analysed and a significant portion of urine samples often contain more than one organism (or a single Gram-positive organism). Only four of the included publications reported tested polymicrobial or mixed sample types. It is important to be aware that the accuracy of AST can be considered on a per-sample level or on a per-isolate level. When samples are tested directly (without isolating individual species from the sample), many methods cannot or do not deliver per-isolate susceptibility or resistance, but only offer per-sample results. There are many studies that assess the accuracy of AST methods on a per-isolate level, but these were out of scope of this review. Any study reporting the accuracy of AST on isolates—but not also reporting accuracy for urine samples—did not meet our inclusion criteria. Most laboratories do not routinely conduct AST for urine samples containing multiple organisms. If mixed growth of bacteria is detected (i.e. two or more species visible on culture plate), the individual isolates may not be routinely separated, these isolates will then not have AST performed and no AST result will be reported for that urine sample. These samples are difficult to interpret and furthermore can be affected by sampling methods and time to culture,^64,65^ which can make the causative pathogen unclear. Given that mixed samples are not often analysed nor antibiotic susceptibilities reported for these samples, we may take single organism datasets as a useful and even complete dataset. In the context of current practice, we can indicate that direct disc diffusion can be accurate if mixed samples are reported as mixed and no AST reported for these samples. However, further research is warranted to better understand how to assess the antibiotic sensitivity for patients with urine containing multiple bacterial species.

Another gap in the evidence is in assessing overall reporting time when the method is embedded in a testing workflow, rather than the method time. While removing an overnight incubation step can without question improve the speed of AST, the overall time taken from the time that a urine sample is collected, to the reporting of clinically actionable results, includes pre-analytical and health system factors. Until overall reporting times can be reduced, it will also be challenging to measure the utility of direct-from-urine AST in terms of improved patient outcomes or antibiotic stewardship. Similarly, health economic assessment will be valuable to ascertain the cost benefits of introducing direct-from-urine AST methods into current clinical workflows.

A limitation to interpreting the accuracy reported by some studies is lack of reported prevalence of resistance. While CA is a commonly used measure of accuracy of AST, the accuracy of tests is calculated as sensitivity and specificity and the ability to determine susceptibility and resistance to different antibiotics. This can be confidently reported when a suitable number of both resistant and susceptible organisms are tested. For observational studies, the prevalence of resistance will depend on the current distribution, and for many antibiotics tested and used empirically this will be low. This can result in very few resistant organisms being available in a randomly selected cohort. For example, nitrofurantoin has extremely low prevalence of resistance (∼1%–10%)^66^ so accuracy assessment for this antibiotic may need to be supplemented by testing samples with known resistant organisms. With direct-from-urine testing, this may not be possible because the resistance of any given sample cannot be determined without 2 days testing, by which time the sample or bacteria composition is likely to have changed. Instead, assessment using simulated samples, with panels of resistant organisms spiked into sterile urine, may be essential.

Furthermore, several of the studies incorrectly calculate major errors (ME) and very major errors (VME). In some studies, VME and ME were calculated based on the number of false resistant or false susceptible results divided by the total number of tests. However, ideally VME and ME should be divided by the total number of correctly identified resistant and susceptible bacteria respectively. While this difference will not affect the OCA, it will artificially lower the error rates determined by these studies, hence why they were not reported in the accuracy measurements.

### Limitations of this scoping review

It is important to acknowledge the methodological limitations of this review. One key consideration is the intentionally broad inclusion criteria, which were designed to capture not only high-quality comparative studies but also those using heterogeneous methodologies, including novel and emerging technologies that may only be designed to demonstrate proof-of-concept. While this approach allowed for a more comprehensive overview of the current landscape, it also introduced variability in study design, sample size and outcome reporting. Many of the included studies featured incomplete datasets, small sample sizes and limited comparisons of antibiotic agents. Consequently, a meta-analysis was not conducted, as the heterogeneity and data limitations would have undermined the validity of any pooled estimates. Furthermore, the review study protocol was not prospectively registered restricting the protocol to be viewed before reporting.

### Conclusions

We present a scoping review that identifies and explores 33 publications that report the accuracy of direct-from-urine AST. Although this represents a substantial and growing body of research, significant heterogeneity and several specific methodological weaknesses limit the opportunity to conduct meta-analysis or compare different AST methods, suggesting that improved study design together with a more standardized evaluation approach may be needed to strengthen the evidence base. More than half of these (18 studies) evaluated a version of a direct urine disc-diffusion method, with this method tested with >3000 urine samples, with all studies reporting CA that exceeds 90%. However, all of these 18 studies were limited in analysis to reporting accuracy only for samples containing a single organism. Together, these reports suggest that a direct-from-urine AST approach has the potential to be accurate, especially when mixed samples are not included or where AST results for mixed organism urine samples are not reported. While other AST methods have also been evaluated, these reports vary greatly in bacterial detection method, sample processing and sample size, and so the evidence of accuracy for other methods is less substantial. Such studies often represent proof-of-concept studies for developing technologies and represent the growing interest in rapid AST for use in UTI prescribing. Overall, there were too many variations in both direct AST methods or protocols and in study design to be able to make any meaningful comparison of the accuracy of different methods within the current review. While these methods or devices still show similar reported levels of CA, further evidence of accuracy, robustness to interfering substances and a wider variety of samples tested would be required to confirm accuracy for these emerging approaches. The widespread uptake of a direct-from-urine AST method would require accuracy of a clearly defined method to be benchmarked against a reference standard method, along with defining clear requirements for other information that is required to best interpret the results, such as organism and mixed culture identification or specific patient groups.
